# LncRNA SNHG14 promotes OGD/R-induced neuron injury by inducing excessive mitophagy via miR-182-5p/BINP3 axis in HT22 mouse hippocampal neuronal cells

**DOI:** 10.1186/s40659-020-00304-4

**Published:** 2020-09-10

**Authors:** Zexiang Deng, Hao Ou, Fei Ren, Yujiao Guan, Ye Huan, Hongwei Cai, Bei Sun

**Affiliations:** 1 Department of Anesthesiology, Xiangya Hospital, Central South University, No. 87 Xiangya Road, Changsha, 410008 Hunan China; 2 Department of Emergency and Critical Care Medicine, The Third Xiangya Hospital, Central South University, Changsha, 410013 Hunan China

**Keywords:** SNHG14, miR-182-5p, BINP3, Mitophagy, Cerebral ischemia–reperfusion injury

## Abstract

**Background:**

Long non-coding RNA (lncRNA) small nucleolar RNA host gene 14 (SNHG14) is associated with cerebral ischemia–reperfusion (CI/R) injury. This work aims to explore the role of SNHG14 in CI/R injury.

**Methods:**

HT22 (mouse hippocampal neuronal cells) cell model was established by oxygen–glucose deprivation/reoxygenation (OGD/R) treatment. The interaction among SNHG14, miR-182-5p and BNIP3 was verified by luciferase reporter assay. Flow cytometry, western blot and quantitative real-time PCR were performed to examine apoptosis, the expression of genes and proteins.

**Results:**

SNHG14 and BNIP3 were highly expressed, and miR-182-5p was down-regulated in the OGD/R-induced HT22 cells. OGD/R-induced HT22 cells exhibited an increase in apoptosis. SNHG14 overexpression promoted apoptosis and the expression of cleaved-caspase-3 and cleaved-caspase-9 in the OGD/R-induced HT22 cells. Moreover, SNHG14 up-regulation enhanced the expression of BNIP3, Beclin-1, and LC3II/LC3I in the OGD/R-induced HT22 cells. Furthermore, SNHG14 regulated BNIP3 expression by sponging miR-182-5p. MiR-182-5p overexpression or BNIP3 knockdown repressed apoptosis in OGD/R-induced HT22 cells, which was abolished by SNHG14 up-regulation.

**Conclusion:**

Our study demonstrates that lncRNA SNHG14 promotes OGD/R-induced neuron injury by inducing excessive mitophagy via miR-182-5p/BINP3 axis in HT22 mouse hippocampal neuronal cells. Thus, SNHG14/miR-182-5p/BINP3 axis may be a valuable target for CI/R injury therapies.

## Background

Cerebral ischemia–reperfusion (CI/R) injury is an extremely complex pathophysiological process, showing a rapid cascade reaction. Cerebral ischemic injury induces the expression of inflammatory cytokines and inflammatory cell infiltration, which in turn causes inflammatory reactions to aggravate brain tissue damage [1]. Reperfusion causes an increase of reactive oxygen species. Reactive oxygen species leads to oxidative stress to promote endothelial dysfunction, DNA damage and local inflammatory response [2]. Inflammatory cascade and oxidative stress may damage cellular structures and lead to cell death [3]. Due to the dependence of cells on mitochondria, the regulation of apoptosis, necrosis and autophagy are closely related to mitochondria [4]. Previous study has shown that mitochondrial damage and dysfunction during CI/R are important causes of nerve cell death [5]. Mitophagy, as a way to clear damaged mitochondria, plays a vital role in CI/R [6], but it still remains controversial. Recent study has reported that electroacupuncture improves CI/R injury by activating Pink1/Parkin-induced mitophagy [7]. Pink1/Parkin-mediated mitophagy ameliorates CI/R injury by eliminating ATF4 and NLRP3 inflammasome [8]. However, Bcl-2/E1B 19 kDa-interacting protein 3 (BNIP3) is highly expressed after CI/R, which causes excessive activation of mitophagy, triggers delayed cell death, and aggravates CI/R injury [9]. These findings show that mitophagy is a double-edged sword. Appropriate mitophagy helps to protect nerve cells, whereas disordered or excessive mitophagy may be harmful to nerve cells. Thus, in-depth study of mitophagy can better analyze the pathological process of CI/R injury.

Our previous study has found that long non-coding RNA (lncRNA) small nucleolar RNA host gene 14 (SNHG14) is highly expressed in CI/R injury and hypoxia-reoxygenation-induced neurons. SNHG14 functions as a competing endogenous RNA to repress miR-30b-5p, which controls its down-stream target Atg5 and Beclin1, thereby enhancing autophagy activity. MiR-182-5p may bind to 3′ untranslated region (UTR) of BNIP3 to repress BNIP3 expression [10]. Whether SNHG14 can affect CI/R injury by regulating miR-182-5p/BNIP3 axis has not been reported.

BNIP3 is a pro-apoptotic mitochondrial protein. BNIP3 and NIX competitively bind to anti-apoptotic Bcl-2, dissociate the Bcl-2/Beclin1 complex and release Beclin1. The released Beclin1 activates autophagy and mitophagy [11]. BNIP3 can independently activates excessive mitophagy leading to cell death [9]. BNIP3 located on damaged mitochondria interacts with LC3-II on neonatal autophagosomes, thereby activating mitophagy [12]. At the same time, BNIP3 also increases mitochondrial dynamin-related protein 1 (Drp1), thereby stimulating the fragmentation of the mitochondrial network and promoting the phagocytosis of damaged mitochondria [13]. Therefore, we speculate that SNHG14 may aggravate neuron injury by inducing excessive mitophagy via miR-182-5p/BINP3 axis.

## Materials and methods

### Cell culture

Mouse hippocampal neuronal cell line (HT22) was obtained from public cell banks (ATCC, Manassas, VA, USA). The cells were cultured in dulbecco’s modified eagle medium (DMEM) (Sangon Biotech, Shanghai, China) supplemented with 10% fetal bovine serum (FBS) and 1% penicillin/streptomycin. And the cells were incubated in a humidified atmosphere at 37 °C and 5% CO_2_.

### Oxygen–glucose deprivation/reoxygenation (OGD/R) application

The cell medium was removed and the HT22 cells were washed with glucose-free DMEM for several times. To initiate oxygen–glucose deprivation (OGD), HT22 cells were cultured in glucose-free DMEM in a tri-gas incubator (Heraeus, Hanau, Germany) for 6 h. The culture conditions were 37 °C, 0.5% O_2_, 94.5% N_2_ and 5% CO_2_. After that, the glucose-free DMEM was removed, and HT22 cells were grown in complete DMEM. The HT22 cells were incubated under 95% air and 5% CO_2_ at 37 °C for 24 h. The culture conditions were simulated the reperfusion. HT22 cells in the control group were treated identically except that they were not exposed to OGD.

### Cell transfection

The SNHG14 was subcloned into the vector pcDNA3.1 (Invitrogen, Carlsbad, CA, USA), generating the vector pcDNA3.1-SNHG14. The negative control (NC) vector pcDNA3.1-NC was used as a control. The miR-182-5p mimic, miR-182-5p inhibitor and the corresponding NC were synthesized by RIBOBIO (Guangzhou, China). Small interfering RNA (siRNA) specific targeting SNHG14 (si-SNHG14) or BNIP3 (si-BNIP3) and its NC were purchased from RIBOBIO. Plasmids were transfected into the cells using Lipofectamine 2000 (Invitrogen) following the manufacturer’s protocol.

### Flow cytometry

Flow cytometry was performed to detect the apoptosis of cells. The cells were collected and washed with pre-cooling PBS for 2 times. Cells were then resuspended in the Annexin V Binding buffer. The cell suspension was dyed with Annexin V-FITC and PI and plunged into darkness at room temperature for 15 min. Then the cell suspension was mixed with Annexin V Binding buffer and put on ice. The apoptosis rate of cells was determined by flow cytometry in an hour. The assay was performed according to the instruction of Annexin V-FITC/PI Cell Apoptosis Detection Kit (TransGen Biotech, Beijing, China).

### Quantitative real-time PCR (QRT-PCR)

QRT-PCR was used to measure the expression intensity of different genes. Total RNA was extracted from cells using TRIzol reagent (Invitrogen). The purity of RNA was detected using NanoDrop 2000 spectrophotometer (Thermo Fisher Scientific, Waltham, MA, USA). The quantity of RNA was determined by agarose gel electrophoresis. The RNA was reversely transcribed to complementary DNA using PrimeScript™ RT Reagent Kit (Takara, Tokyo, Japan). QRT-PCR was carried out using SYBR Green PCR Mix Kit (Takara) according to the instruction. The results were analyzed using the ∆∆CT (cycle threshold) method for quantification. β-actin was used as an internal control. Primer sequences used in this study were as follows: SNHG14: forward: 5′-GGGTGTTTACGTAGACCAGAACC-3′, reverse: 5′-CTTCCAAAAGCCTTCTGCCTTAG-3′; miR-182-5p: forward: 5′-TTAGGAACCCTCCTCTCTC-3′, reverse: 5′-CGGTGATGTGAAGAAGGA-3′; BNIP3: forward: 5′-AACTCAGATTGGATATGGGATTGG-3′, reverse: 5′-AGAGCAGCAGAGATGGAAGG-3′; β-actin: forward: 5′-GTCGTACCACAGGCATTGTGTAGG-3′, reverse: 5′-GCAATGCCTGGGTACATGGTGG-3′.

### Western blot (WB)

Total protein was extracted from cells using Tissue or Cell Total Protein Extraction Kit (Sangon Biotech). Equivalent protein from different samples was separated by protein electrophoresis, following by transformation onto PVDF membranes (Merck Millipore, Billerica, MA, USA). The membranes were incubated with the anti-rabbit caspase-3, cleaved-caspase-3, caspase-9, cleaved-caspase-9, PINK-1, Parkin, BNIP3, Beclin-1 and LC3A/B (1:1000, Cell Signaling Technology, Danvers, MA, USA) at 4 °C overnight after immersed into sealed liquid. After the membranes were washed with TBST for several times, goat anti-mouse IgG antibody (1:1000, Cell Signaling Technology) labeled with horseradish peroxidase were incubated with the membranes as a secondary antibody. Anti-mouse β-actin antibody (1:1000, Cell Signaling Technology) was used as a reference protein for normalization. The gray levels of the protein bands were examined by Image J software.

### Luciferase reporter assay

SNHG14 or BNIP3 containing the predicted miR-182-5p binding sites were cloned into pGL3-SNHG14-Wt (wild-type), pGL3-SNHG14-Mut (mutant type), pGL3-BNIP3-Wt or pGL3-BNIP3-Mut vectors (RIBOBIO), respectively. The miR-182-5p mimic and mimic NC were synthesized by RIBOBIO. The Wt (Mut) SNHG14 vector or Wt (Mut) 3′UTR of BNIP3 vector and miR-182-5p mimic or mimic NC were co-transfected into 293T cells by using Lipofectamine 2000 Transfection Reagent (Invitrogen). The luciferase activity of the cells was detected after 48 h of transfection using the luciferase assay system (Ambion, Austin, TX, USA).

### Statistical analysis

All experiments were independently repeated at least 3 times. All values were exhibited as mean ± standard deviation and analyzed by SPSS 22.0 statistical software (IBM, Armonk, NY, USA). For comparison of two groups, a two-tailed Student’s t test was used. Comparison of multiple groups was made using a one- or two-way ANOVA. Difference was considered statistically significant at *P* < 0.05.

## Results

### SNHG14 overexpression promotes apoptosis of OGD/R-induced HT22 cells

**Fig. 1:**
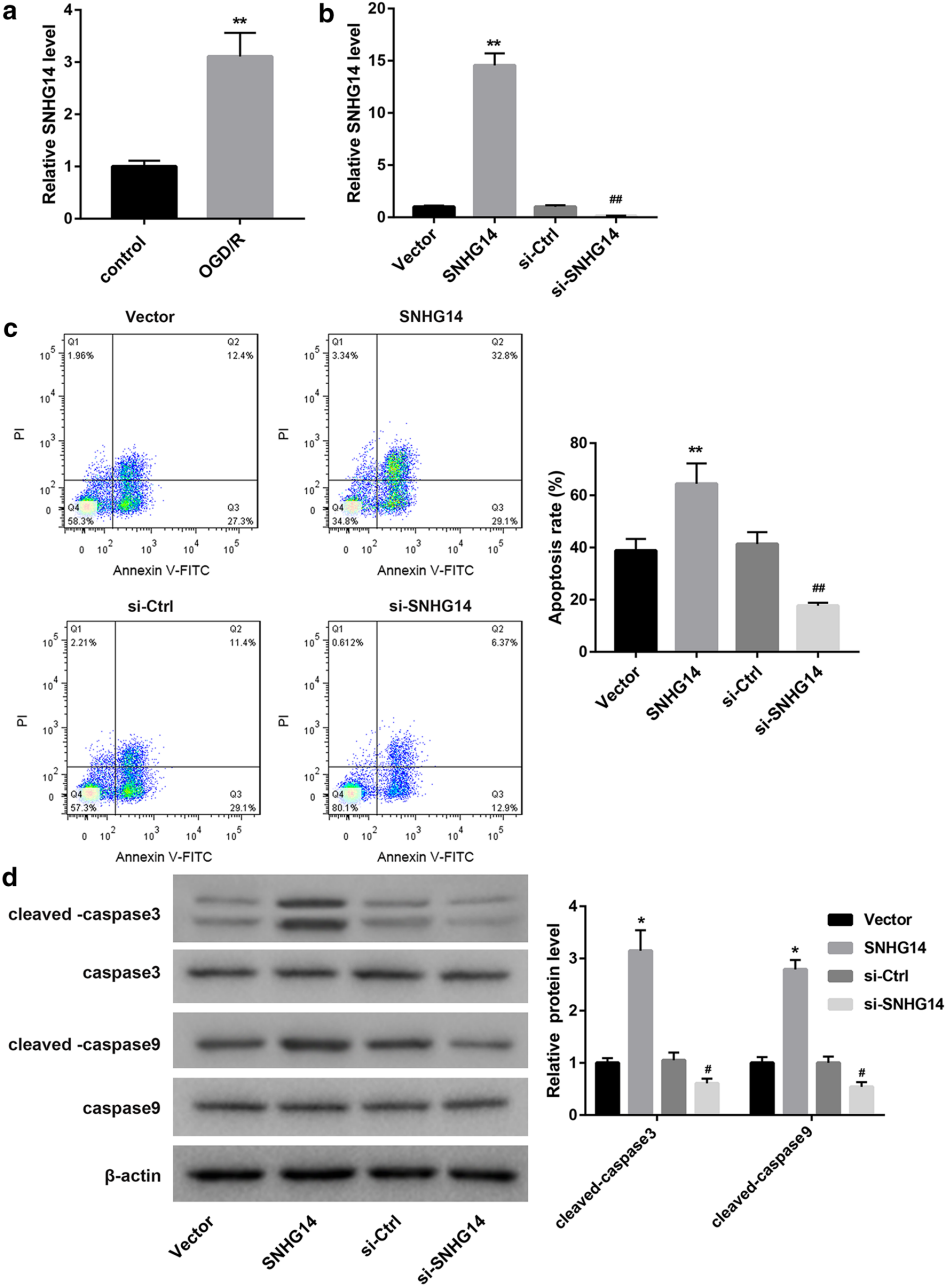
SNHG14 overexpression promotes apoptosis of OGD/R-induced HT22 cells. HT22 cell model was established by OGD/R treatment. Normal HT22 cells served as control. **a** QRT-PCR was performed to assess the expression of SNHG14 in the HT22 cells. HT22 cells were transfected with pcDNA3.1-SNHG14, pcDNA3.1-NC, si-SNHG14 or si-Ctrl. Then, the modified HT22 cells were subjected to OGD/R treatment. **b** QRT-PCR was performed to detect the expression of SNHG14 in the modified HT22 cells. **c** Flow cytometry was performed to explore the apoptosis of the modified HT22 cells. **d** The expression of caspase-3, cleaved-caspase-3, caspase-9 and cleaved-caspase-9 in the modified HT22 cells was examined by WB. (*P < 0.05, **P < 0.01, versus Control or Vector; ^#^*P* < 0.05, ^##^*P* < 0.01, versus si-Ctrl)

To investigate the role of SNHG14 in cerebral ischemia–reperfusion injury, HT22 cells were subjected to OGD/R treatment. We assessed the expression of SNHG14 in the HT22 cells by qRT-PCR, showing that SNHG14 was highly expressed in the OGD/R-induced HT22 cells (Fig. 1a). Then, we estimated apoptosis of the HT22 cells by flow cytometry. Compared with control HT22 cells, OGD/R-induced HT22 cells displayed an increase in apoptosis (Additional file 1: Figure S1). Furthermore, HT22 cells were transfected with pcDNA3.1-SNHG14 or si-SNHG14 to induce SNHG14 overexpression or knockdown in HT22 cells, and then the modified HT22 cells were subjected to OGD/R treatment. We found that SNHG14 up-regulation notably enhanced the expression of SNHG14 in the HT22 cells, whereas SNHG14 knockdown significantly repressed the expression of SNHG14 in the HT22 cells (Fig. 1b). Moreover, the apoptosis of the modified HT22 cells was estimated by flow cytometry. SNHG14 overexpression caused a boost in apoptosis of HT22 cells. The apoptosis of HT22 cells was severely inhibited by SNHG14 silencing (Fig. 1c). In addition, the expression of caspase-3, cleaved-caspase-3, caspase-9 and cleaved-caspase-9 in the HT22 cells was examined by WB. As show in Fig. 1d, SNHG14 overexpression and SNHG14 knockdown have no effect on the expression of caspase-3 and caspase-9 in the HT22 cells. The expression of cleaved-caspase-3 and cleaved-caspase-9 in the HT22 cells was notably increased in the HT22 cells after transfected with pcDNA3.1-SNHG14. SNHG14 silencing led to a decrease in the expression of cleaved-caspase-3 and cleaved-caspase-9 in the HT22 cells. Therefore, these data suggest that SNHG14 overexpression promotes apoptosis of OGD/R-induced HT22 cells.

### SNHG14 overexpression promotes mitophagy of OGD/R-induced HT22 cells via BNIP3/Beclin-1 signaling pathway

**Fig. 2:**
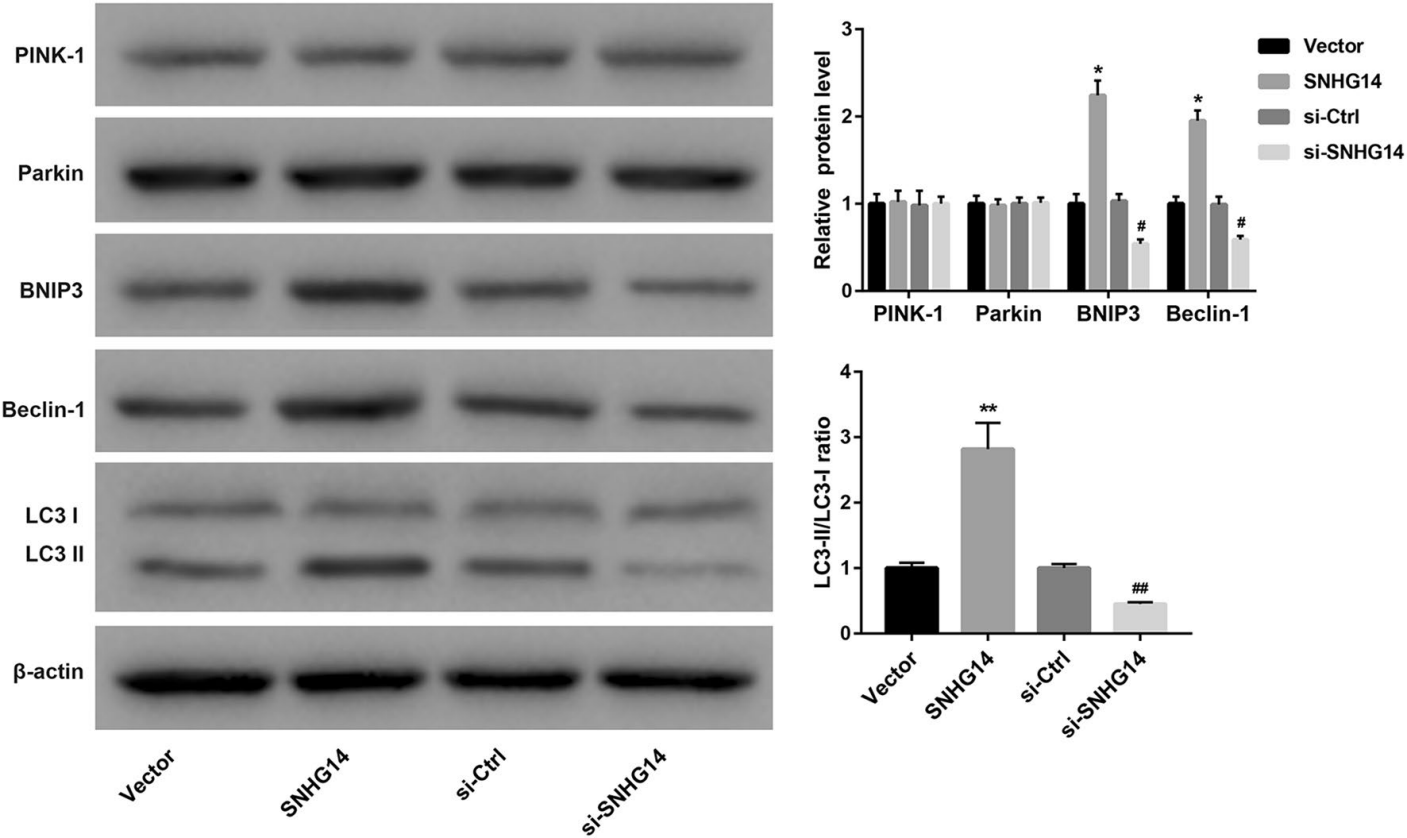
SNHG14 overexpression promotes mitophagy of OGD/R-induced HT22 cells. HT22 cells were transfected with pcDNA3.1-SNHG14, pcDNA3.1-NC, si-SNHG14 or si-Ctrl. Then, the modified HT22 cells were subjected to OGD/R treatment. The expression of PINK-1, Parkin, BNIP3, Beclin-1, LC3I and LC3II in the modified HT22 cells was examined by WB. (*P < 0.05, **P < 0.01, versus Vector; ^#^*P* < 0.05, ^##^*P* < 0.01, versus si-Ctrl)

Next, we investigated the effect of SNHG14 overexpression or knockdown on mitophagy of OGD/R-induced HT22 cells. The expression of mitophagy-related proteins in the HT22 cells was detected by WB. Figure 2 shows that SNHG14 overexpression or knockdown has no effect on the expression of PINK-1 and Parkin in the HT22 cells, suggesting that SNHG14 could not activate PINK-1/Parkin signaling pathway to induce mitophagy. Moreover, SNHG14 overexpression led to an increase of BNIP3 and Beclin-1 expression, whereas SNHG14 knockdown caused a decrease of BNIP3 and Beclin-1 expression in the HT22 cells. LC3II/LC3I ratio was notably enhanced by SNHG14 up-regultaion in the HT22 cells. SNHG14 silencing significantly repressed LC3II/LC3I ratio in the HT22 cells (Fig. 2). Taken together, these findings indicate that SNHG14 overexpression promotes mitophagy of OGD/R-induced HT22 cells via BNIP3/Beclin-1 signaling pathway.

### SNHG14 regulates BNIP3 expression by sponging miR-182-5p

**Fig. 3:**
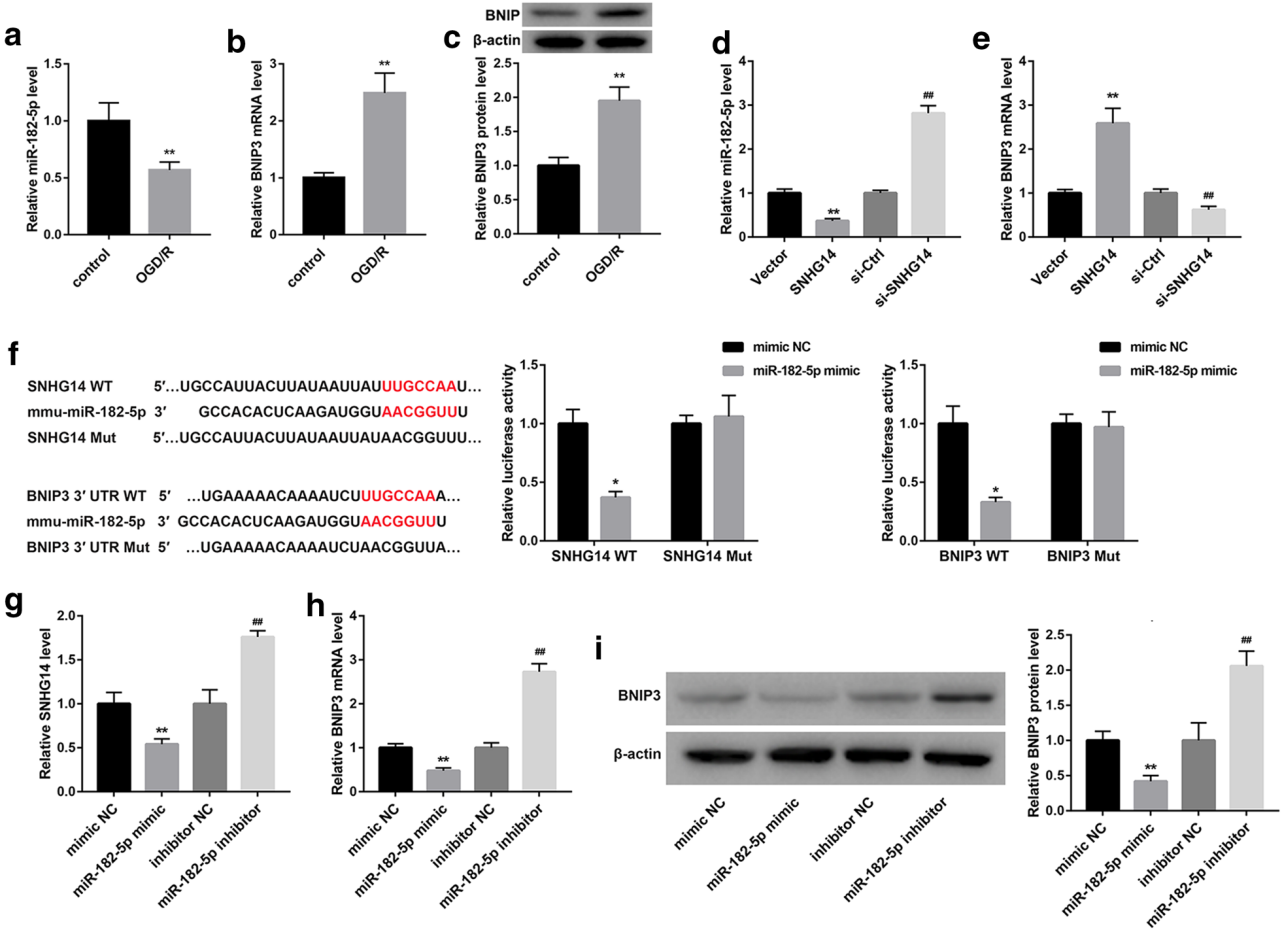
SNHG14 regulates BNIP3 expression by sponging miR-182-5p. HT22 cell model was established by OGD/R treatment. Normal HT22 cells served as control. **a** QRT-PCR was performed to assess the expression of miR-182-5p in the HT22 cells. **b**, **c** QRT-PCR and WB were performed to estimate the expression of BNIP3 in the HT22 cells. HT22 cells were transfected with pcDNA3.1-SNHG14, pcDNA3.1-NC, si-SNHG14 or si-Ctrl. **d**, **e** QRT-PCR was performed to detect the expression of miR-182-5p and BNIP3 in the modified HT22 cells. **f** The Wt (Mut) SNHG14 vector or Wt (Mut) BNIP3 vector and miR-182-5p mimic or mimic NC were co-transfected into 293T cells. The interaction among SNHG1, miR-182-5p and BNIP3 was measured by luciferase reporter assay. HT22 cells were transfected with miR-182-5p mimic, mimic NC, miR-182-5p inhibitor or inhibitor NC. **g** QRT-PCR was performed to assess the expression of SNHG14 in the modified HT22 cells. **h**, **i** The gene and protein expression of BNIP3 in the modified HT22 cells was explored by qRT-PCR or WB. (*P < 0.05, **P < 0.01, versus Vector or mimic NC; ^#^*P* < 0.05, ^##^*P* < 0.01, versus si-Ctrl or inhibitor NC)

To explore the molecular mechanism of SNHG14 in cerebral ischemia–reperfusion injury, we examined the expression of miR-182-5p and BNIP3 in the OGD/R-induced HT22 cells. Compared with control HT22 cells, OGD/R-induced HT22 cells exhibited a decrease of miR-182-5p expression (Fig. 3a). The gene and protein expression of BNIP3 was notably up-regulated in the HT22 cells after OGD/R treatment (Fig. 3b, c). Furthermore, HT22 cells were transfected with pcDNA3.1-SNHG14, pcDNA3.1-NC, si-SNHG14 or si-Ctrl, the expression of miR-182-5p and BNIP3 in the modified HT22 cells was assessed by qRT-PCR and WB. The expression of miR-182-5p in the HT22 cells was severely suppressed by SNHG14 up-regulation and notably enhanced by SNHG14 down-regulation (Fig. 3d). SNHG14 overexpression significantly promoted BNIP3 expression, whereas SNHG14 silencing notably repressed BNIP3 expression in the HT22 cells (Fig. 3e). Bioinformatics predicts that SNHG14 binds to miR-182-5p. To verify this hypothesis, we performed luciferase reporter assay to further investigate the relationship among SNHG14, miR-182-5p and BNIP3. The data showed that miR-182-5p was the target gene of SNHG14 and BNIP3 was the target gene of miR-182-5p (Fig. 3f). In addition, HT22 cells were transfected with miR-182-5p mimic or miR-182-5p inhibitor to induce miR-182-5p up-regulation or down-regulation in the HT22 cells. The gene or protein expression of SNHG14 and BNIP3 in the HT22 cells was detected by qRT-PCR and WB. MiR-182-5p overexpression caused a pronounced decrease of SNHG14 expression, whereas miR-182-5p silencing led to a boost of SNHG14 expression in the HT22 cells (Fig. 3g). The gene and protein expression of BNIP3 were severely repressed by miR-182-5p up-regulation and markedly enhanced by miR-182-5p knockdown in the HT22 cells (Fig. 3h, i). Thus, these data taken together confirm that SNHG14 functions as a competing endogenous RNA to repress miR-182-5p, which controls its down-stream target BNIP3.

### SNHG14 overexpression promotes mitophagy of OGD/R-induced HT22 cells via miR-182-5p/BNIP3 axis

**Fig. 4:**
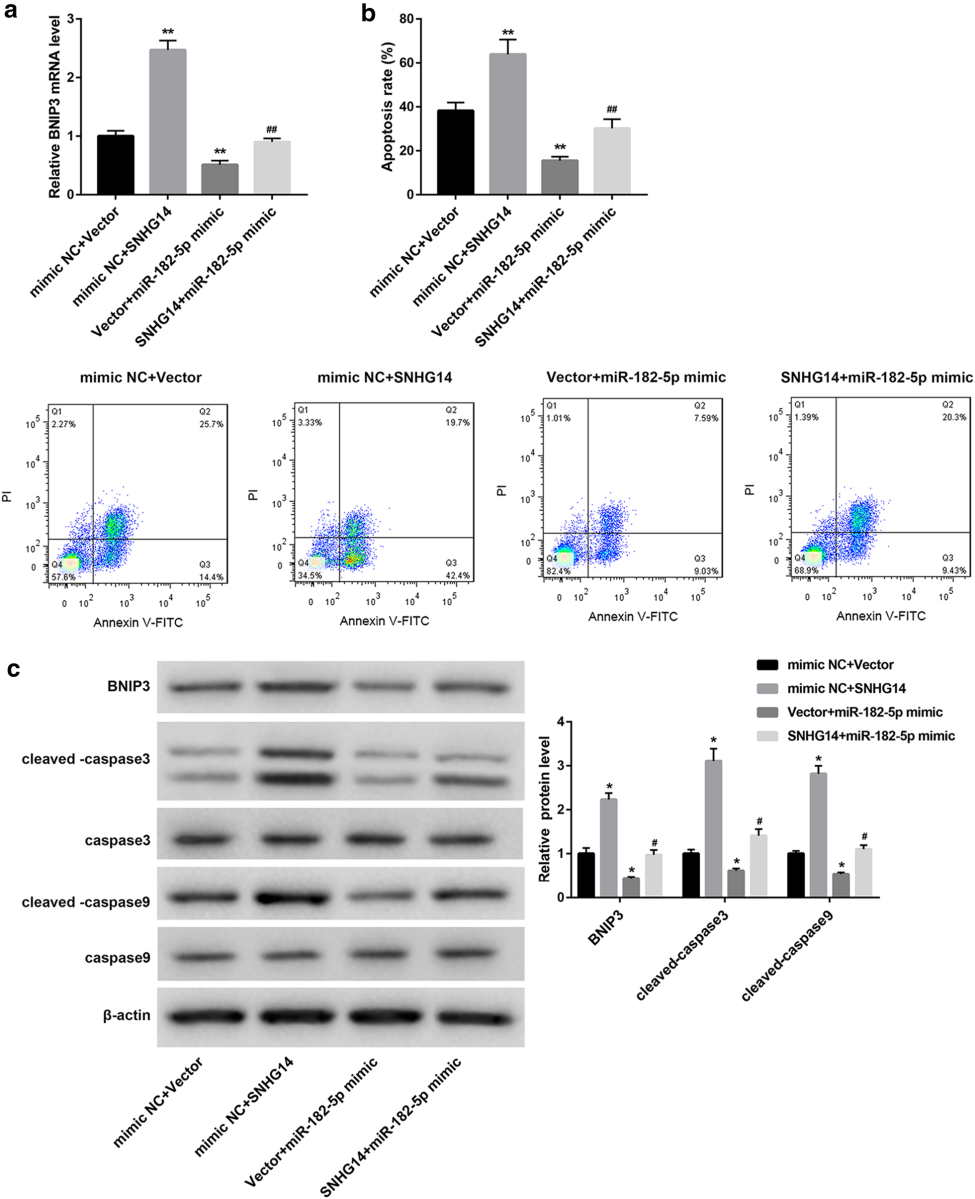

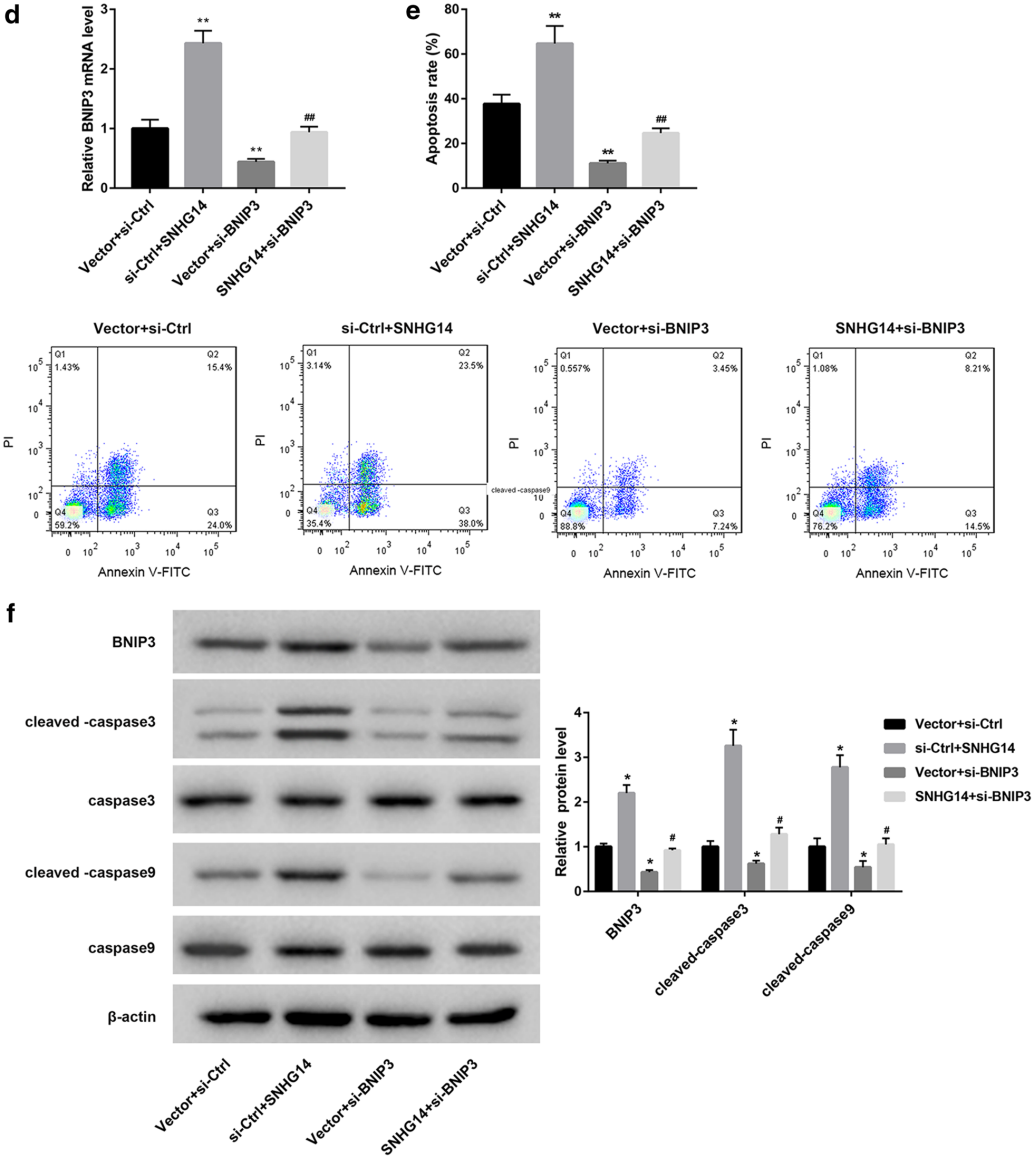
SNHG14 overexpression promotes mitophagy of OGD/R-induced HT22 cells via miR-182-5p/BNIP3 axis. PcDNA3.1-SNHG14 or pcDNA3.1-NC and miR-182-5p mimic or mimic NC were co-transfected into HT22 cells. Then, the modified HT22 cells were subjected to OGD/R treatment. **a** QRT-PCR was performed to detect the expression of BNIP3 in the modified HT22 cells. **b** Flow cytometry was performed to explore the apoptosis of the modified HT22 cells. **c** The expression of BNIP3, caspase-3, cleaved-caspase-3, caspase-9 and cleaved-caspase-9 in the modified HT22 cells was examined by WB. PcDNA3.1-SNHG14 or pcDNA3.1-NC and si-BNIP3 or si-Ctrl were co-transfected into HT22 cells. Then, the modified HT22 cells were subjected to OGD/R treatment. **d** QRT-PCR was performed to detect the expression of BNIP3 in the modified HT22 cells. **e** Flow cytometry was performed to explore the apoptosis of the modified HT22 cells. **f** The expression of BNIP3, caspase-3, cleaved-caspase-3, caspase-9 and cleaved-caspase-9 in the modified HT22 cells was examined by WB. (*P < 0.05, **P < 0.01, versus mimic NC + Vector or Vector + si-Ctrl; ^#^*P* < 0.05, ^##^*P* < 0.01, versus Vector + miR-182-5p mimic Vector + si-BNIP3)

To verify the mechanism of SNHG14 in regulating mitophagy, pcDNA3.1-SNHG14 or pcDNA3.1-NC and miR-182-5p mimic or mimic NC were co-transfected into HT22 cells. Then, the HT22 cells were subjected to OGD/R treatment, and we detected the expression of BNIP3 in the modified HT22 cells. SNHG14 overexpression remarkably enhanced BNIP3 expression, whereas miR-182-5p up-regulation notably repressed BNIP3 expression in the HT22 cells. The inhibiting effect of miR-182-5p overexpression on BNIP3 expression in the HT22 cells was rescued by SNHG14 up-regulation (Fig. 4a). Furthermore, we performed flow cytometry to explore apoptosis of the HT22 cells. SNHG14 overexpression enhanced apoptosis of HT22 cells. MiR-182-5p overexpression led to a pronounced decrease in apoptosis of the HT22 cells, which was abolished by SNHG14 overexpression (Fig. 4b). Moreover, the expression of BNIP3, caspase-3, cleaved-caspase-3, caspase-9, and cleaved-caspase-9 in the HT22 cells was examined by WB. As shown in Fig. 4c, there is no significant difference in the expression of caspase-3 and caspase-9 among the four groups. The expression of BNIP3, cleaved-caspase-3 and cleaved-caspase-9 in the HT22 cells is notably enhanced by SNHG14 overexpression, whereas the expression of BNIP3, cleaved-caspase-3 and cleaved-caspase-9 in the HT22 cells was severely decreased by miR-182-5p overexpression. The influence conferred by miR-182-5p overexpression was abolished by SNHG14 up-regulation. In addition, pcDNA3.1-SNHG14 or pcDNA3.1-NC and si-BNIP3 or si-Ctrl was co-transfected into HT22 cells. Then, the HT22 cells were subjected to OGD/R treatment, and qRT-PCR was performed to detect the expression of BNIP3 in the HT22 cells. SNHG14 overexpression notably promoted the expression of BNIP3 in the HT22 cells, whereas BNIP3 silencing significantly suppressed the expression of BNIP3 in the HT22 cells. The inhibiting effect of BNIP3 silencing on BNIP3 expression in the HT22 cells was rescued by SNHG14 up-regulation (Fig. 4d). Then, we performed flow cytometry to explore apoptosis of the HT22 cells. SNHG14 up-regulation significantly promoted apoptosis of the HT22 cells. BNIP3 silencing caused a severe decrease in apoptosis of the HT22 cells, which was abolished by SNHG14 overexpression (Fig. 4e). Subsequently, the expression of BNIP3, caspase-3, cleaved-caspase-3, caspase-9, and cleaved-caspase-9 in the HT22 cells was examined by WB. We found that SNHG14 up-regulation and BNIP3 knockdown had no influence on the expression of caspase-3 and caspase-9 in the HT22 cells. SNHG14 up-regulation significantly promoted BNIP3, cleaved-caspase-3 and cleaved-caspase-9 expression in the HT22 cells, and these proteins were severely down-regulated in the HT22 cells after BNIP3 silencing. The influence conferred by BNIP3 knockdown was rescued by SNHG14 up-regulation (Fig. 4f). Therefore, these data demonstrate that SNHG14 overexpression promotes mitophagy of OGD/R-induced HT22 cells by regulating miR-182-5p/BNIP3.

## Discussion

Studies over the past two decades have provided important information on the role of SNHG14 in various cancers. The expression of SNHG14 is up-regulated in various cancers, such as osteosarcoma [14], pancreatic ductal adenocarcinoma [15], and lung adenocarcinoma [16]. SNHG14 exerts oncogenic functions in these cancers and promotes the progression of cancers. Surveys such as that conducted by Zhao et al. have shown that SNHG14 is highly expressed in non-small cell lung cancer cells. SNHG14 participates in the cell proliferation, invasion and migration of non-small cell lung cancer cells by regulating miR-206/G6PD [17]. As an oncogene in prostate cancer, SNHG14 contributes to the progression of prostate cancer by sponging miR-613 [18]. Recent study has confirmed that SNHG14 is up-regulated in middle cerebral artery occlusion/reperfusion treated brain tissues and OGD/R-treated PC-12 cells [19]. In our study, we found that SNHG14 was highly expressed in OGD/R-induced HT22 cells. OGD/R treatment induced apoptosis of HT22 cells. SNHG14 overexpression further enhanced apoptosis and the expression of apoptosis-related proteins, cleaved-caspase-3 and cleaved-caspase-9, in the OGD/R-induced HT22 cells. SNHG14 silencing had the opposite results. Thus, these findings show that SNHG14 overexpression promotes apoptosis of OGD/R-induced HT22 cells.

Previous study has revealed that SNHG14 enhances inflammatory response induced by CI/R injury [19]. However, our work found that SNHG14 may be associated with mitophagy in CI/R injury. The PINK1/Parkin signaling pathway is the most typical signaling pathway that regulates mitophagy. PINK1/Parkin-mediated mitophagy has a crucial role in various diseases, such as CI/R injury [20], Parkinson’s disease and septic acute kidney injury [21, 22]. However, SNHG14 overexpression or knockdown had no effect on the expression of PINK1 and Parkin, suggesting that SNHG14 can not regulate mitophagy by activating PINK1/Parkin signaling pathway. In addition, the expression of BNIP3 and Beclin-1 in the OGD/R-induced HT22 cells was enhanced by SNHG14 overexpression and suppressed by SNHG14 silencing. SNHG14 up-regulation enhanced LC3II/LC3I ratio, whereas SNHG14 knockdown repressed LC3II/LC3I ratio in the OGD/R-induced HT22 cells. Under hypoxic microenvironment, up-regulated BNIP3 competitively binds Bcl-2, dissociates the Bel-2/Beclin-1 complex and release Beclin-1 to activate mitophagy [23]. And in the case of myocardial ischemia–reperfusion injury, BNIP3 also activates mitophagy to clear damaged mitochondria [24]. LC3II and LC3I are autophagy-related proteins, and LC3II specifically binds to newly synthesized autophagosomes. Thus, the ratio of LC3II/LC3I is closely associated with mitophagy. Therefore, our work suggests that SNHG14 overexpression promotes mitophagy of OGD/R-induced HT22 cells.

Previous studies have confirmed that miR-182-5p is highly expressed in bladder cancer and unprotected left main coronary artery disease, and miR-182-5p could be a diagnostic biomarkers for unprotected left main coronary artery disease [25, 26]. In our study, we found that miR-182-5p was down-regulated, and BNIP3 was up-regulated in OGD/R-induced HT22 cells. MiR-182-5p was the target gene of SNHG14, and BNIP3 was the target gene of miR-182-5p. Moreover, SNHG14 suppressed miR-182-5p expression and enhanced BNIP3 expression. Therefore, our findings suggest that SNHG14 functions as a competing endogenous RNA for miR-182-5p, thereby leading to the upregulation of the activity of its endogenous target, BNIP3. Furthermore, miR-182-5p overexpression led to a pronounced decrease in apoptosis of the HT22 cells, which was abolished by SNHG14 overexpression. SNHG14 up-regulation promoted apoptosis of the HT22 cells, and apoptosis were notably suppressed by BNIP3 knockdown. And the influence conferred by BNIP3 knockdown was rescued by SNHG14 overexpression. As a member of the Bcl-2 family of cell death-regulating factors, BNIP3 plays a crucial role in mitochondrial dysfunction and mitophagy homeostasis, and BNIP3 has become a potential therapeutic target for diseases of secondary mitochondrial dysfunction [27]. BNIP3 protects against renal ischemia–reperfusion injury by regulating mitophagy [28]. PAR-4 regulates autophagic cell death in human malignant glioma by up-regulating p53 and BNIP3 expression [29]. Deferoxamine treatment combined with sevoflurane postconditioning improves myocardial ischemia reperfusion injury by regulating HIF-1/BNIP3-mediated mitophagy in diabetic rats [30]. Thus, these data taken together reveal that SNHG14 overexpression promotes mitophagy of OGD/R-induced HT22 cells by regulating miR-182-5p/BNIP3 axis.

## Conclusions

In summary, our studies demonstrate that LncRNA SNHG14 promotes OGD/R-induced neuron injury by inducing excessive mitophagy via miR-182-5p/BINP3 axis in HT22 mouse hippocampal neuronal cells. Thus, our findings suggest that the SNHG14/miR-182-5p/BINP3 axis could be a valuable target for CI/R injury therapies.

## Supplementary information

**Additional file 1:** Figure S1. OGD/R-induced HT22 cells exhibit an increase in apoptosis. HT22 cell model was established by OGD/R treatment. Normal HT22 cells served as control. Flow cytometry was performed to explore the apoptosis of the HT22 cells. (^**^P < 0.01, versus Control).

## Data Availability

The datasets used and/or analysed during the current study are available from the corresponding author on reasonable request.

## Abbreviations

BNIP3: Bcl-2/E1B 19 kDa-interacting protein 3
CI/R: Cerebral ischemia–reperfusion
DMEM: Dulbecco’s modified eagle medium
Drp1: Dynamin-related protein 1
FBS: Fetal bovine serum
lncRNA: Long non-coding RNA
Mut: Mutant type
NC: Negative control
OGD: Oxygen-glucose deprivation
OGD/R: Oxygen-glucose deprivation/reoxygenation
qRT-PCR: Quantitative real-time PCR
siRNA: Small interfering RNA
SNHG14: Small nucleolar RNA host gene 14
UTR: Untranslated region
WB: Western blot
Wt: Wild-type
