# Supplementary figures and images for: LncRNA SNHG14 promotes OGD/R-induced neuron injury by inducing excessive mitophagy via miR-182-5p/BINP3 axis in HT22 mouse hippocampal neuronal cells
Source: Biol Res. 2020 Sep 10;53:38. doi: 10.1186/s40659-020-00304-4 (PMC7488096; doi:10.1186/s40659-020-00304-4)

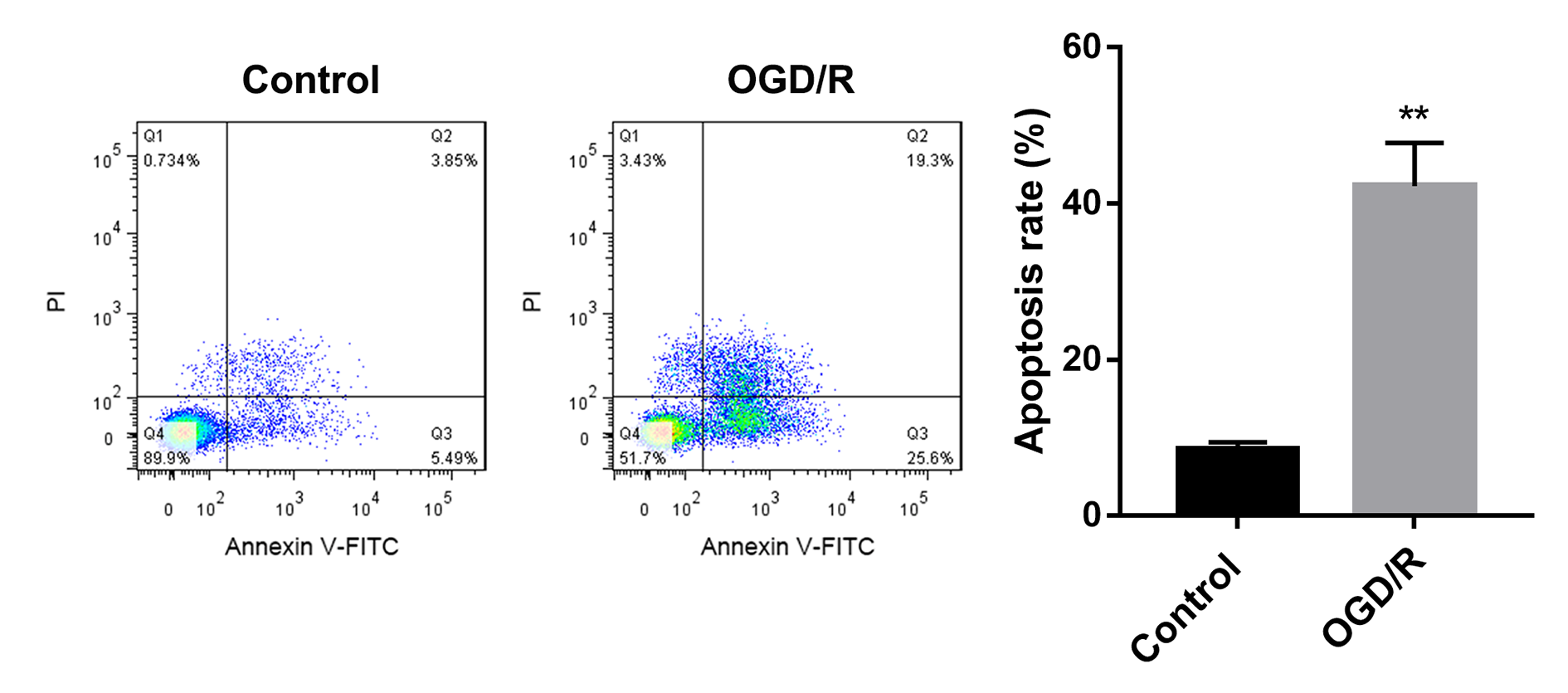

Supplement: Supplementary file 1 — Additional file 1: Figure S1. OGD/R-induced HT22 cells exhibit an increase in apoptosis. HT22 cell model was established by OGD/R treatment. Normal HT22 cells served as control. Flow cytometry was performed to explore the apoptosis of the HT22 cells. (**P < 0.01, versus Control). [file 40659_2020_304_MOESM1_ESM.tif]
